# Relationship between dietary patterns and overactive bladder: a cross sectional study of NHANES 2013 to 2023

**DOI:** 10.3389/fnut.2025.1554794

**Published:** 2025-05-13

**Authors:** Yiming Ding, Yaru Mou, Dongming Wang, Zeyong Niu, Pengge Xin, Yu Zhou, Guoxin Song, Hongjia Xu, Jian Wang

**Affiliations:** ^1^Department of Pediatric Surgery, Qilu Hospital of Shandong University, Jinan, China; ^2^Cheeloo College of Medicine, Shandong University, Jinan, China; ^3^Department of Cardiology, Shandong Provincial Hospital Affiliated to Shandong First Medical University, Jinan, China; ^4^Department of Pediatric Surgery, Weihai Municipal Hospital, Weihai, China; ^5^Department of Neurology, The Second Hospital, Cheeloo College of Medicine, Shandong University, Jinan, China

**Keywords:** overactive bladder (OAB), dietary patterns, principal component analysis (PCA), dietary risk factors, bladder health

## Abstract

**Background:**

Dietary patterns, as a comprehensive dietary indicator, may influence the risk of developing overactive bladder (OAB). However, it remains unclear whether dietary patterns independently affect the development of OAB.

**Objective:**

This study aimed to identify specific dietary patterns using principal component analysis (PCA) and evaluate their associations with the risk of OAB, thereby providing new insights for OAB prevention and management.

**Methods:**

Dietary patterns were identified by applying PCA, and their associations with OAB risk were analyzed. After adjusting for three known confounders (age, sex, and BMI), four key dietary patterns were determined: (1) PC5: Antioxidant-balanced pattern, OR = 0.96, 95% CI (0.94–0.97), *p* < 0.05; (2) PC16: Diversified low-alcohol pattern, OR = 0.92, 95% CI (0.89–0.94), *p* < 0.05; (3) PC18: Whole-grain high-alcohol pattern, OR = 1.04, 95% CI (1.01–1.07), *p* < 0.05; (4) PC22: High-fiber low-sugar pattern, OR = 0.93, 95% CI (0.90–0.96), *p* < 0.05.

**Results:**

The findings indicated that the antioxidant-balanced (PC5), diversified low-alcohol (PC16), and high-fiber low-sugar (PC22) dietary patterns were associated with a decreased risk of OAB, while the whole-grain high-alcohol pattern (PC18) exhibited a dual effect. Specifically, when considered independently, the high-fiber low-sugar pattern showed a protective effect; however, when combined with the whole-grain high-alcohol pattern, it increased the risk of OAB.

**Conclusion:**

Dietary patterns are independent factors influencing the development of OAB. In particular, the antioxidant-balanced, diversified low-alcohol, and high-fiber low-sugar patterns help reduce OAB risk, whereas the whole-grain high-alcohol pattern exerts a dual effect.

## 1 Introduction

Overactive bladder (OAB) typically refers to the abnormal contraction of the bladder during the storage phase in the absence of urinary tract infections or structural bladder abnormalities. OAB is commonly characterized by symptoms such as urgency, frequency, increased nocturia, and/or urinary incontinence. In the United States, OAB affects a considerable portion of the population, with a prevalence of 17% in men and 30% in women (1). OAB significantly impairs quality of life, disrupts intimate relationships, and may even lead to depression or anxiety (1), making the investigation of its risk factors highly relevant. Recent studies have identified several factors associated with OAB. For example, a meta-analysis by Lin Zhang and colleagues, which reviewed nearly two decades of literature, indicated that women, the elderly, and obese individuals are more prone to developing OAB (2).

As an important lifestyle factor, a healthy dietary pattern is increasingly considered beneficial for lower urinary tract function (3). Different populations often exhibit significant variations in their dietary patterns; for example, obese individuals tend to have high-fat and high-sugar diets, while low-protein, low-calorie diets are more common among the elderly (4). Currently, it remains inconclusive whether dietary patterns independently influence the development of OAB or if their effects are mediated indirectly through factors such as gender, age, and obesity. Additionally, among these three confounders, there is no consensus on the indicator used to describe obesity. Based on the research by Hannestad et al. (5). Hannestad et al. (5) we have chosen BMI as the measure to characterize patients’ obesity levels.

At the molecular level, there may be a deeper connection between OAB and dietary patterns. Studies have shown that overactive bladder induced by diabetes (6) and metabolic syndrome (MetS) is associated with oxidative stress or autonomic neuropathy (7), potentially involving the activation of the NF-κB signaling pathway (8). This process may include oxidative stress responses, such as the significant upregulation of Superoxide Dismutase (SOD) gene expression, suggesting that the body is attempting to counteract damage from free radicals (9). Certain foods may play a significant role in reducing inflammation, scavenging free radicals, and suppressing oxidative stress. For instance, some studies have indicated that the phenolic compounds found in certain berries possess antioxidant properties and can exert positive effects on inflammatory diseases. Moreover, not only specific foods but also dietary patterns rich in natural antioxidants (10), such as the Mediterranean diet, have been shown to help maintain lower urinary tract function. Notably, according to the findings of Nani et al. (11), the Mediterranean diet exhibits potential in antioxidant and anti-inflammatory functions due to its high polyphenol content. Numerous studies have now demonstrated that dietary patterns can affect both the incidence and severity of OAB, thereby providing the initial theoretical basis for this study. However, research directly proving the association between certain dietary patterns and OAB as risk factors remains limited, with a lack of definitive interventional studies. Therefore, the aim of this study is to use principal component analysis (PCA) to extract and summarize dietary patterns in the population and to demonstrate that dietary patterns are independent risk factors for OAB.

Principal component analysis (PCA) extracts the main components from dietary data, enabling the study to focus not on specific nutrients but on the overall dietary preferences of the sample. PCA offers several advantages: compared with factor analysis, PCA is more direct and interpretable; and compared with cluster analysis, PCA emphasizes the intrinsic relationships among variables rather than simple grouping, which is more conducive to revealing the true characteristics of the dietary structure (12).

As mentioned earlier, current methods for exploring the association between diet and OAB typically begin by assuming that certain dietary factors—such as specific foods (e.g., berries) or existing dietary patterns (e.g., the Mediterranean diet)—are related to OAB, and then use statistical methods to prove this association (13). This approach relies on the researcher’s subjective assumptions rather than objective extraction of the overall dietary trends from existing data.

Furthermore, the principal components derived from PCA can be directly used in subsequent regression analyses, making it more suitable for exploring the quantitative relationship between dietary patterns and disease. Using PCA to extract principal components is a common method in studying population dietary patterns. Applying PCA to identify dietary patterns and analyze their association with OAB risk is both a reasonable and important approach in practice.

The main purpose of this study is to investigate whether dietary patterns are independent factors influencing the development of OAB, thereby providing scientific evidence for dietary interventions in OAB management.

## 2 Materials and methods

### 2.1 Analytical overview

After cleaning the NHANES 2013–2023 data, we retained 26,800 adults. All 68 dietary principal components (PCs) produced by PCA were included in the initial screening. Step 1: Each PC was compared between the OAB and non-OAB groups using an independent samples t-test. PCs with *p* < 0.05 were flagged, and the Bonferroni threshold (0.05 / 68 ≈ 0.00074) was reported alongside. Step 2 (Model 1): All flagged PCs were entered into a multivariable logistic model to calculate unadjusted odds ratios (ORs) and 95% confidence intervals (CIs). Step 3 (Model 2): PCs that remained significant were re-estimated after adjusting for age, sex, and BMI. Step 4 (Model 3): Among these, only PC5, PC16, PC18, and PC22—whose nutrient loadings align with recognized OAB mechanisms (e.g., antioxidant activity, alcohol exposure, dietary fiber)—were retained to build the final simplified model. A receiver operating characteristic (ROC) analysis showed that predictive performance remained virtually unchanged after simplification, indicating that the stepwise filtering preserved model efficacy. The dual reporting of conventional *p*-values and the Bonferroni threshold, coupled with mechanistic validation, strikes a balance between controlling false positives and retaining meaningful signals, thus enhancing reproducibility. Finally, to explore mixture and non-linear effects, we summarized the four retained PCs into a weighted quantile sum (WQS) index and applied restricted cubic splines (RCS) to provide an integrated, shape-flexible view of dietary influences on OAB risk.

### 2.2 Data sources

This study utilized data from the 2013–2023 National Health and Nutrition Examination Survey (NHANES), a nationally representative, stratified, multistage probability-sampled cross-sectional survey. The following NHANES modules were integrated:

- Kidney and Urinary Conditions: Included overactive bladder (OAB)-related variables such as urinary incontinence and nocturia.
- Dietary Data: 24-h dietary recall interviews focusing on specific nutrient intakes (e.g., carbohydrates, fats, proteins, vitamins). Questions with >80% completion rates were selected. Weighted averages were calculated using Day 1 and Day 2 interviews to reduce bias.
- Demographic Data: Age, sex, and BMI data were matched to cleaned samples based on demographic variables.

### 2.3 Inclusion and exclusion criteria

**Figure 1:**
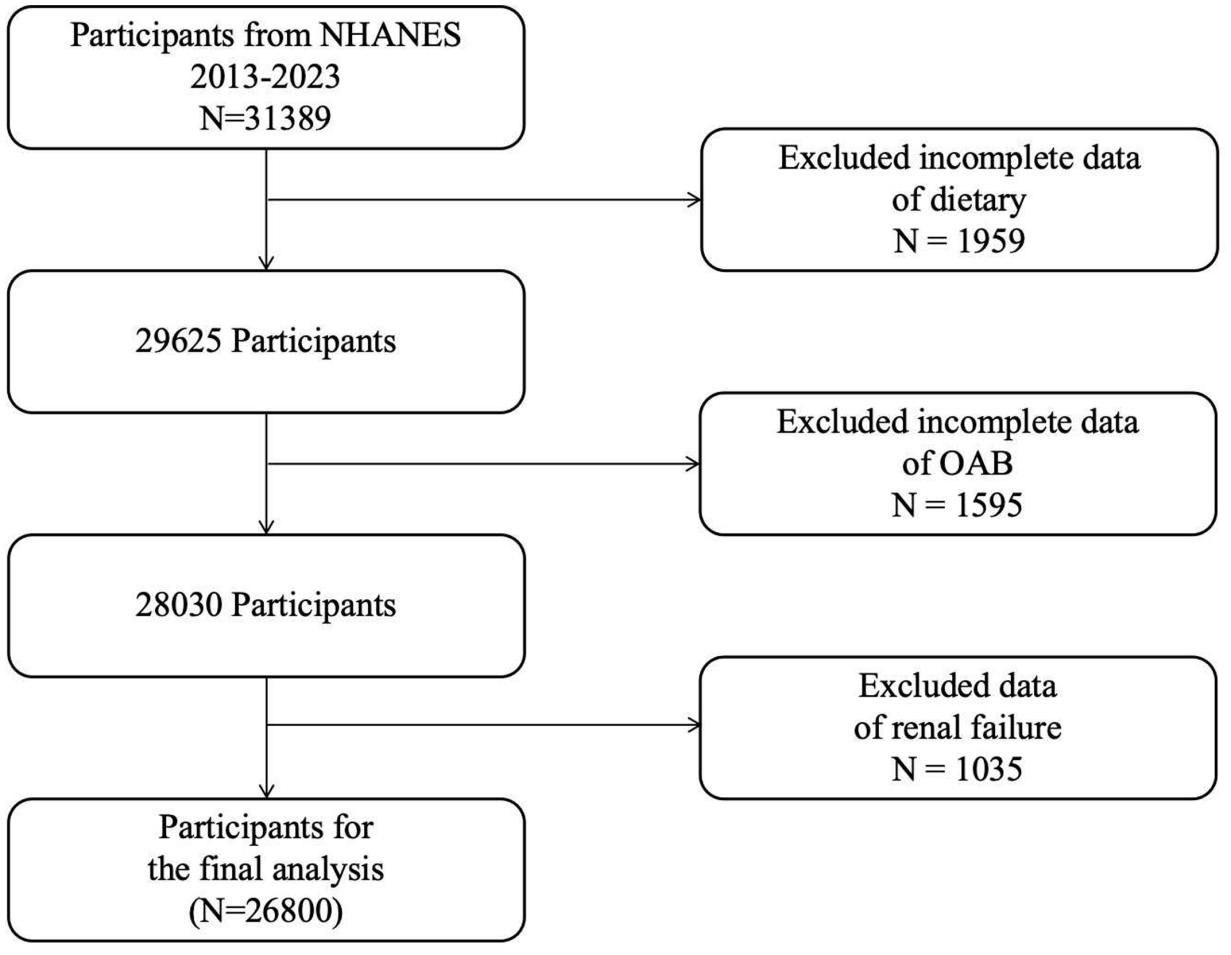
Flowchart depicting the sample selection process from NHANES 2009–2018.

Inclusion Criteria (see Figure 1):

- Adults aged ≥18 years (due to significant differences in metabolic characteristics and urinary control mechanisms between minors and adults; this study focuses on adult OAB).
- Participants with complete kidney/urinary condition questionnaires and 24-h dietary recall data.

Exclusion Criteria:

- Samples with incomplete 24-h dietary recall questionnaires.
- Participants missing kidney/urinary-related questionnaires.
- Patients with renal failure (identified through NHANES question KIQ022, which documents professional diagnosis of renal failure), as abnormal urine output in renal failure may confound OAB diagnosis.

Post-cleaning data handling:

After applying the above criteria, the overall missingness rate was <5%. Missing values in the cleaned dataset were addressed via multiple imputation:

Continuous variables: Imputed with mean values.
Categorical variables: Imputed with mode values.

### 2.4 Operational definition of OAB

OAB status was determined based on responses to NHANES questionnaire items:

KIQ044: “Have you experienced involuntary urine leakage in the past 12 months?”
KIQ480: “In the past 30 days, how many times per night did you typically wake up to urinate?”

Participants were classified as having OAB if they met either of the following criteria:

KIQ044 = 1 (presence of urinary incontinence).
KIQ480 ≥ 2 (nocturia ≥2 episodes per night).

Those not meeting either criterion were assigned to the non-OAB group. Compared to existing literature (14–16), this is a simplified OABSS diagnostic criterion, primarily intended for this study, which applied a strict Bonferroni correction. While this helps control type I error, it may also increase the risk of type II error. Therefore, a more lenient diagnostic standard was used to avoid introducing additional type II errors.

## 3 Statistical analysis

### 3.1 Principal component analysis (PCA)

**Figure 2:**
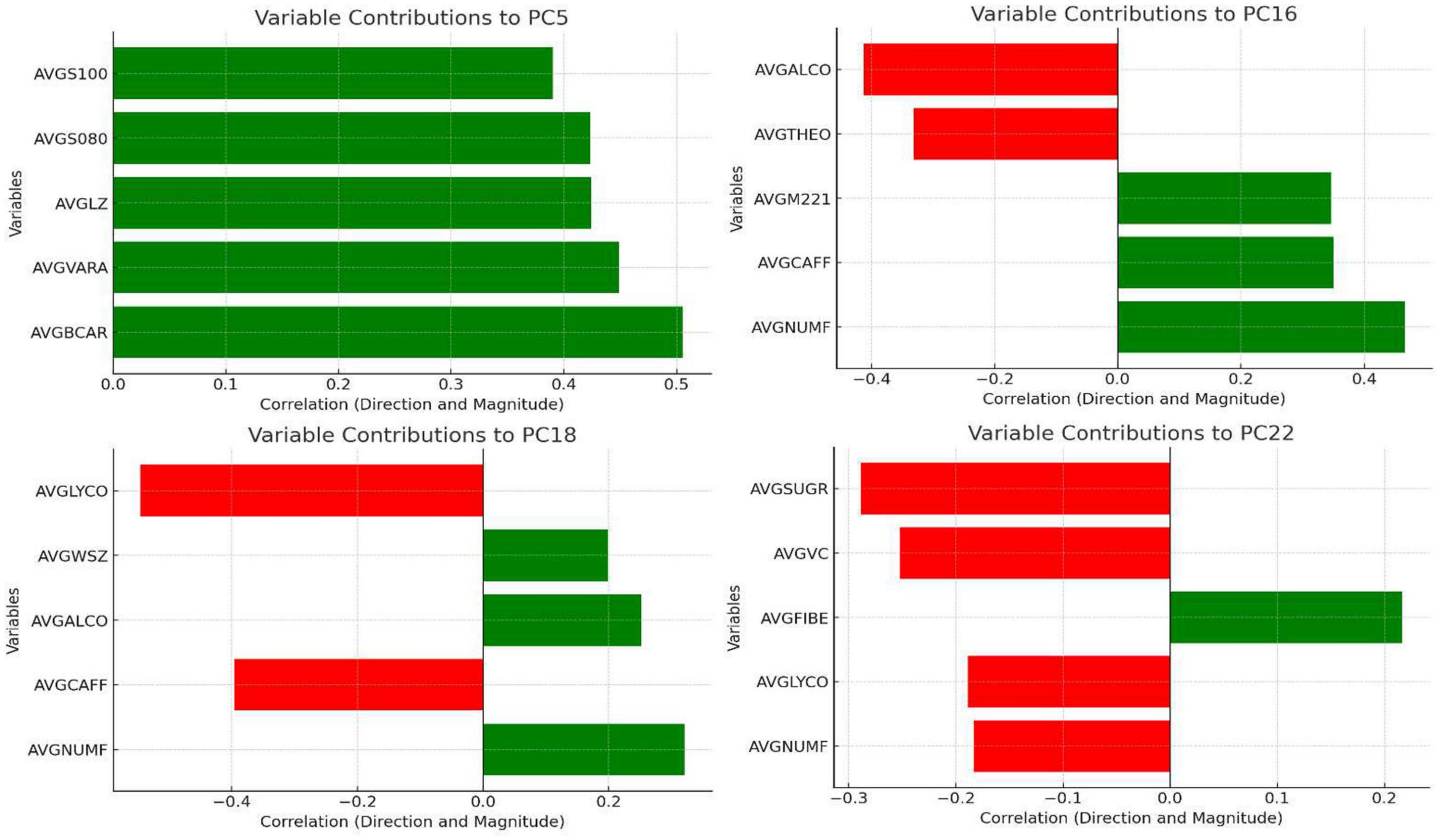
Loading distribution of dietary principal components.

Principal component analysis (PCA), a dimensionality reduction method, was applied to identify major sources of variation among dietary variables and generate components representing overall dietary patterns. A total of 68 principal components were extracted. Each component reflects the proportional contribution (termed “loading”) of different nutrients, as illustrated in Figure 2 for the four key dietary patterns highlighted in this study (see below for selection criteria).

### 3.2 Descriptive statistics

Population characteristics (age, sex, and BMI) and PCA-derived dietary components were summarized. Baseline comparisons between the OAB and non-OAB groups for categorical variables were performed using chi-square tests (χ^2^). For variable selection, we initially adopted a relatively lenient criterion and then applied a more stringent criterion in the final refined model. Specifically, we performed t-tests on 68 principal components (PCs), using *p* < 0.05 as the inclusion threshold to capture as many potentially OAB-related components as possible and avoid omitting important factors. Meanwhile, we also referred to the Bonferroni-corrected significance threshold for these 68 PCs (approximately 0.05/68 ≈ 0.00074) and used 0.00074 as an approximate benchmark reflecting the Bonferroni principle.

t-tests to screen dietary components significantly associated with OAB (Table 1).

**Table 1:** Baseline characteristics analysis.

Variable	Overall mean (SD)	OAB mean (SD)	Non-OAB mean (SD)	*p*-value
Age	51.25 (17.52)	57.23 (16.29)	45.88 (16.84)	0.000
Age group: youth	6,435 (100%)	1,688 (26.23%)	4,747 (73.77%)	
Age group: middle-aged	10,843 (100%)	4,751 (43.82%)	6,092 (56.18%)	
Age group: elderly	9,522 (100%)	6,239 (65.52%)	3,283 (34.48%)	
Gender:1	12,832 (100%)	5,290 (41.23%)	7,542 (58.77%)	
Gender:2	13,968 (100%)	7,388 (52.89%)	6,580 (47.11%)	
BMI	29.83 (7.36)	30.89 (7.79)	28.88 (6.82)	0.000
BMI group: obese	11,075 (100%)	6,004 (54.21%)	5,071 (45.79%)	
BMI group: non-obese	15,725 (100%)	6,674 (42.44%)	9,051 (57.56%)	
Dietary PC1	0.00 (5.51)	−0.23 (5.38)	0.20 (5.61)	0.000
Dietary PC2	0.00 (2.10)	0.01 (2.07)	0.01 (2.13)	0.321
Dietary PC3	0.00 (1.94)	0.02 (1.90)	0.02 (1.97)	0.099
Dietary PC4	0.00 (1.70)	0.05 (1.68)	0.04 (1.71)	0.000
Dietary PC5	0.00 (1.63)	0.06 (1.61)	0.05 (1.65)	0.000
Dietary PC6	0.00 (1.41)	0.03 (1.61)	0.03 (1.20)	0.000
Dietary PC7	0.00 (1.25)	0.04 (1.25)	0.04 (1.26)	0.000
Dietary PC8	0.00 (1.20)	0.00 (1.13)	0.00 (1.25)	0.950
Dietary PC9	0.00 (1.14)	0.05 (1.16)	0.00 (1.25)	0.000
Dietary PC10	0.00 (1.09)	0.02 (1.05)	0.04 (1.12)	0.010
Dietary PC11	0.00 (1.06)	0.02 (1.05)	0.02 (1.06)	0.007
Dietary PC12	0.00 (1.05)	0.02 (1.03)	0.02 (1.07)	0.043
Dietary PC13	0.00 (1.01)	−0.02 (1.01)	0.02 (1.01)	0.006
Dietary PC14	0.00 (1.01)	0.01 (0.96)	0.01 (1.04)	0.063
Dietary PC15	0.00 (0.98)	0.00 (0.96)	0.00 (1.00)	0.569
Dietary PC16	0.00 (0.95)	0.02 (0.95)	0.00 (0.96)	0.000
Dietary PC17	0.00 (0.95)	0.01 (0.85)	0.00 (1.03)	0.192
Dietary PC18	0.00 (0.92)	0.01 (0.85)	0.01 (0.93)	0.000
Dietary PC19	0.00 (0.90)	0.03 (0.91)	0.01 (0.92)	0.084
Dietary PC20	0.00 (0.87)	0.01 (0.87)	0.00 (0.88)	0.057
Dietary PC21	0.00 (0.86)	0.01 (0.86)	0.00 (0.88)	0.535
Dietary PC22	0.00 (0.85)	0.01 (0.86)	0.00 (0.85)	0.000
Dietary PC23	0.00 (0.79)	−0.03 (0.78)	0.03 (0.81)	0.095
Dietary PC24	0.00 (0.74)	0.01 (0.72)	0.01 (0.75)	0.939
Dietary PC25	0.00 (0.69)	0.00 (0.67)	0.00 (0.71)	0.000
Dietary PC26	0.00 (0.66)	−0.02 (0.85)	0.02 (0.68)	0.121
Dietary PC27	0.00 (0.63)	−0.01 (0.63)	0.01 (0.63)	0.003
Dietary PC28	0.00 (0.62)	0.01 (0.58)	0.00 (0.62)	0.571
Dietary PC29	0.00 (0.60)	0.01 (0.56)	0.00 (0.62)	0.000
Dietary PC30	0.00 (0.60)	0.01 (0.52)	0.00 (0.61)	0.288
Dietary PC31	0.00 (0.58)	0.00 (0.47)	0.00 (0.59)	0.000
Dietary PC32	0.00 (0.57)	0.01 (0.46)	0.01 (0.57)	0.186
Dietary PC33	0.00 (0.56)	0.00 (0.43)	0.00 (0.56)	0.476
Dietary PC34	0.00 (0.55)	0.00 (0.41)	0.00 (0.55)	0.000
Dietary PC35	0.00 (0.54)	0.01 (0.39)	0.00 (0.54)	0.000
Dietary PC36	0.00 (0.53)	0.00 (0.37)	0.00 (0.52)	0.007
Dietary PC37	0.00 (0.52)	0.00 (0.36)	0.00 (0.51)	0.456
Dietary PC38	0.00 (0.51)	0.00 (0.34)	0.00 (0.50)	0.555
Dietary PC39	0.00 (0.50)	0.00 (0.33)	0.00 (0.48)	0.040
Dietary PC40	0.00 (0.49)	0.00 (0.32)	0.00 (0.47)	0.246
Dietary PC41	0.00 (0.48)	0.00 (0.30)	0.00 (0.46)	0.919
Dietary PC42	0.00 (0.47)	0.00 (0.29)	0.00 (0.45)	0.025
Dietary PC43	0.00 (0.46)	0.00 (0.28)	0.00 (0.44)	0.019
Dietary PC44	0.00 (0.45)	0.00 (0.27)	0.00 (0.43)	0.038
Dietary PC45	0.00 (0.44)	0.00 (0.27)	0.00 (0.42)	0.457
Dietary PC46	0.00 (0.43)	0.00 (0.26)	0.00 (0.41)	0.066
Dietary PC47	0.00 (0.42)	0.00 (0.24)	0.00 (0.40)	0.524
Dietary PC48	0.00 (0.41)	0.00 (0.23)	0.00 (0.39)	0.000
Dietary PC49	0.00 (0.40)	0.00 (0.22)	0.00 (0.38)	0.025
Dietary PC50	0.00 (0.39)	0.00 (0.22)	0.00 (0.37)	0.063
Dietary PC51	0.00 (0.38)	0.00 (0.20)	0.00 (0.36)	0.309
Dietary PC52	0.00 (0.37)	0.00 (0.19)	0.00 (0.35)	0.025
Dietary PC53	0.00 (0.36)	0.00 (0.18)	0.00 (0.34)	0.250
Dietary PC54	0.00 (0.35)	0.00 (0.17)	0.00 (0.33)	0.000
Dietary PC55	0.00 (0.34)	0.00 (0.16)	0.00 (0.32)	0.580
Dietary PC56	0.00 (0.33)	0.00 (0.15)	0.00 (0.31)	0.151
Dietary PC57	0.00 (0.32)	0.00 (0.14)	0.00 (0.14)	0.489
Dietary PC58	0.00 (0.31)	0.00 (0.14)	0.00 (0.14)	0.087
Dietary PC59	0.00 (0.30)	0.00 (0.14)	0.00 (0.12)	0.001
Dietary PC60	0.00 (0.29)	0.00 (0.12)	0.00 (0.10)	0.349
Dietary PC61	0.00 (0.28)	0.00 (0.10)	0.00 (0.04)	0.154
Dietary PC62	0.00 (0.10)	0.00 (0.04)	0.00 (0.03)	0.000
Dietary PC63	0.00 (0.04)	0.00 (0.03)	0.00 (0.03)	0.293
Dietary PC64	0.00 (0.03)	0.00 (0.02)	0.00 (0.03)	0.221
Dietary PC65	0.00 (0.02)	0.00 (0.02)	0.00 (0.02)	0.010
Dietary PC66	0.00 (0.01)	0.00 (0.01)	0.00 (0.01)	0.303
Dietary PC67	0.00 (0.01)	0.00 (0.01)	0.00 (0.01)	0.442
Dietary PC68	0.00 (0.00)	0.00 (0.00)	0.00 (0.00)	0.029

### 3.3 Logistic regression analysis

To explore associations between dietary components and OAB, three logistic regression models were constructed:

Model 1 (Univariate): ORs (95% CI) for each dietary component individually.
Model 2 (Multivariate): Adjusted for age, sex, and BMI to calculate ORs (95% CI).
Model 3 (Simplified): Only components retaining significance after adjustment (PC5, PC16, PC18, PC22; *p* < 0.05) were included. ORs (95% CI) are shown in Table 2.

**Table 2:** Multivariable logistic regression analysis.

Component	Model 1 OR	95% CI Model 1	*p*-value Model 1	Model 2 OR	95% CI Model 2	*p*-value Model 2	Model 3 OR	95% CI Model 3	*p*-value Model 3
Age	-	–	–	1.04	1.04–1.05	0.000	1.04	1.04–1.05	0.000
Gender	-	–	–	1.74	1.65–1.84	0.000	1.72	1.63–1.81	0.000
BMI	-	–	–	1.04	1.04–1.04	0.000	1.04	1.04–1.04	0.000
Dietary PC1	0.98	0.98–0.99	0.000	1.01	1.00–1.01	0.029	-	-	-
Dietary PC4	0.97	0.96–0.99	0.000	0.99	0.97–1.01	0.234	-	-	-
Dietary PC5	1.05	1.03–1.06	0.000	0.96	0.94–0.97	0.000	0.96	0.94–0.97	0.000
Dietary PC6	1.04	1.02–1.06	0.000	1.01	0.99–1.03	0.289	-	-	-
Dietary PC7	1.05	1.03–1.08	0.000	0.99	0.97–1.02	0.584	-	-	-
Dietary PC9	1.08	1.06–1.10	0.000	1.01	0.99–1.04	0.250	-	-	-
Dietary PC10	0.97	0.95–0.99	0.011	1.00	0.98–1.02	0.935	-	-	-
Dietary PC11	1.03	1.01–1.05	0.020	-	-	-	-	-	-
Dietary PC12	1.03	1.01–1.05	0.025	-	-	-	-	-	-
Dietary PC13	0.97	0.94–0.99	0.006	1.01	0.98–1.04	0.489	-	-	-
Dietary PC16	1.05	1.03–1.08	0.000	0.92	0.89–0.94	0.000	0.92	0.89–0.94	0.000
Dietary PC18	1.07	1.04–1.11	0.000	1.04	1.01–1.07	0.005	1.04	1.01–1.07	0.005
Dietary PC22	0.9	0.87–0.93	0.000	0.92	0.89–0.96	0.000	0.93	0.90–0.96	0.000
Dietary PC25	0.92	0.89–0.95	0.000	1.00	0.96–1.04	0.844	-	-	-
Dietary PC27	1.07	1.03–1.11	0.001	1.08	1.04–1.13	0.000	-	-	-
Dietary PC29	1.08	1.03–1.13	0.001	1.04	0.99–1.09	0.127	-	-	-
Dietary PC31	1.1	1.05–1.06	0.000	0.99	0.94–1.05	0.806	-	-	-
Dietary PC34	0.88	0.83–0.94	0.000	0.95	0.89–1.01	0.127	-	-	-
Dietary PC35	0.86	0.81–0.92	0.000	0.98	0.92–1.05	0.607	-	-	-
Dietary PC36	0.91	0.86–0.98	0.007	0.95	0.88–1.02	0.132	-	-	-
Dietary PC39	1.08	1.01–1.17	0.034	1.08	1.01–1.08	0.060	-	-	-
Dietary PC42	0.91	0.84–0.99	0.029	0.97	0.94–1.05	0.573	-	-	-
Dietary PC43	0.9	0.82–0.98	0.019	1.00	1.02–1.07	0.940	-	-	-
Dietary PC44	1.11	1.01–1.22	0.030	1.00	0.96–1.04	0.999	-	-	-
Dietary PC48	0.79	0.71–0.89	0.000	0.96	0.95–1.04	0.443	-	-	-
Dietary PC49	1.14	1.02–1.27	0.024	0.96	0.94–1.09	0.474	-	-	-
Dietary PC52	0.86	0.76–0.98	0.021	0.83	0.94–1.03	0.006	-	-	-
Dietary PC54	0.77	0.66–0.89	0.000	0.92	0.94–1.03	0.287	-	-	-
Dietary PC55	0.7	0.58–0.86	0.001	0.81	0.28–14.85	0.049	-	-	-
Dietary PC62	0.15	0.07–0.31	0.000	0.28	0.13–0.6	0.001	-	-	-
Dietary PC65	-	-	-	-	-	-	-	-	-
Dietary PC68	-	-	-	-	-	-	-	-	-

**Figure 3:**
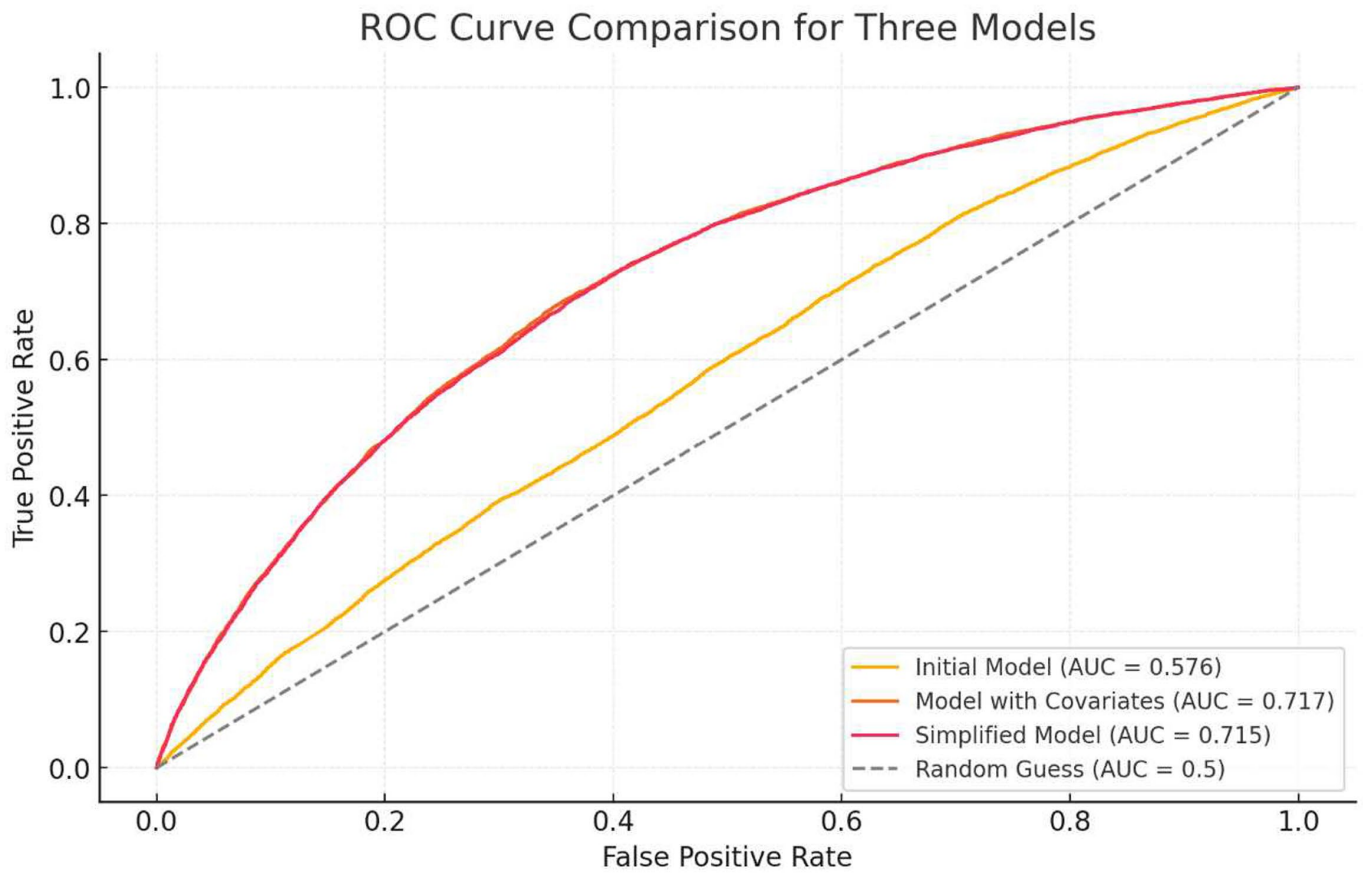
ROC curve of the regression model.

AUC curves were calculated to evaluate model predictive performance (Figure 3).

### 3.4 Principal component independence testing

To address potential multicollinearity, variance inflation factors (VIFs) were calculated for PCA components. A VIF > 5 indicates significant collinearity requiring further adjustment. Results confirmed the independence of the four dietary patterns (Table 3).

**Table 3:** Multicollinearity diagnostics.

Variable	VIF
Dietary_PC5	1
Dietary_PC16	1
Dietary_PC18	1
Dietary_PC22	1

### 3.5 Subgroup analysis

Subgroup analyses were stratified by:

Age (≤50 vs. >50 years),
Sex (male vs. female),
BMI (normal weight vs. overweight vs. obese).

Only components with protective effects (PC5, PC16, PC22) were analyzed to ensure robustness. Results (Table 4) validated the stability and consistency of dietary patterns across subgroups.

**Table 4:** Subgroup analysis.

Subgroup	OR 95%CI	*p*-value
PC5		
Age(Youth)	1.01 (0.97,1.04)	0.7100
Age(Middle-age)	0.98 (0.96,1.00)	0.0790
Age(Elderly)	1.00 (0.97,1.02)	0.8220
BMI(Obese)	1.07 (1.04,1.09)	0.0000
BMI(non-Obese)	1.04 (1.02,1.05)	0.0000
Gender(Male)	1.05 (1.02,1.07)	0.0000
Gender(Female)	0.99 (0.96,1.01)	0.1940
PC16		
Age(Youth)	0.91 (0.85,0.94)	0.0000
Age(Middle-age)	0.96 (0.92,1)	0.0350
Age(Elderly)	0.96 (0.91,1)	0.0420
BMI(Obese)	1.06 (1.01,1.1)	0.0120
BMI(non-Obese)	1.05 (1.02,1.09)	0.0020
Gender(Male)	1.06 (1.03,1.1)	0.0000
Gender(Female)	1.01 (0.97,1.05)	0.6130
PC22		
Age(Youth)	0.91 (0.85,0.98)	0.0100
Age(Middle-age)	0.90 (0.86,0.94)	0.0000
Age(Elderly)	0.95 (0.98,1)	0.0610
BMI(Obese)	0.89 (0.85,0.094)	0.0000
BMI(non-Obese)	0.91 (0.87,0.94)	0.0000
Gender(Male)	0.92 (0.88,0.96)	0.0000
Gender(Female)	0.88 (0.84,0.93)	0.0000

### 3.6 Restricted cubic spline (RCS) analysis

**Figure 4:**
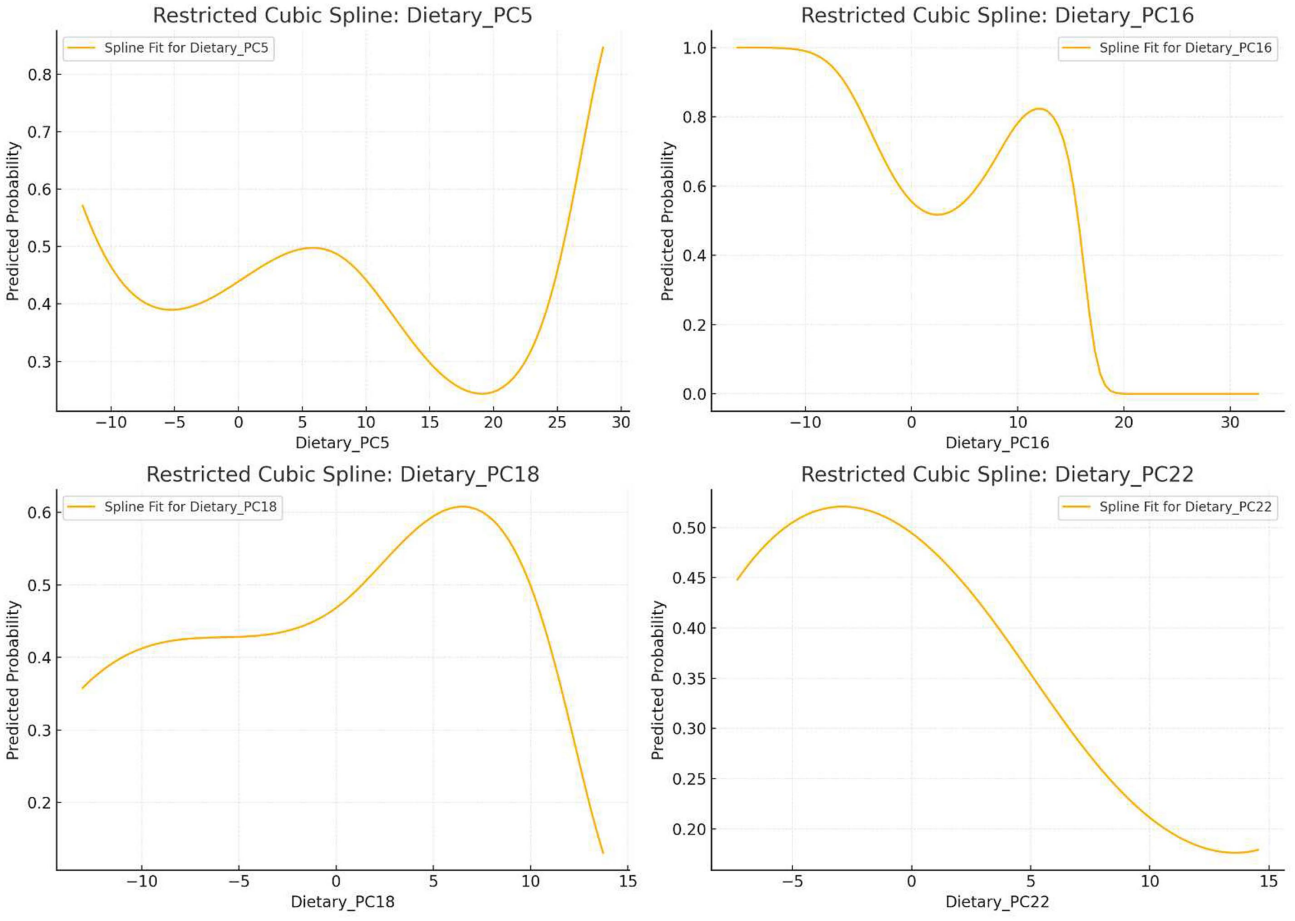
Nonlinear relationship between principal components and OAB incidence.

Nonlinear relationships between PC5, PC16, PC18, PC22, and OAB risk were assessed using 3-knot RCS terms within logistic regression. Trend curves for OAB probability across component scores are shown in Figure 4.

### 3.7 Weighted quantile sum (WQS) model

**Figure 5:**
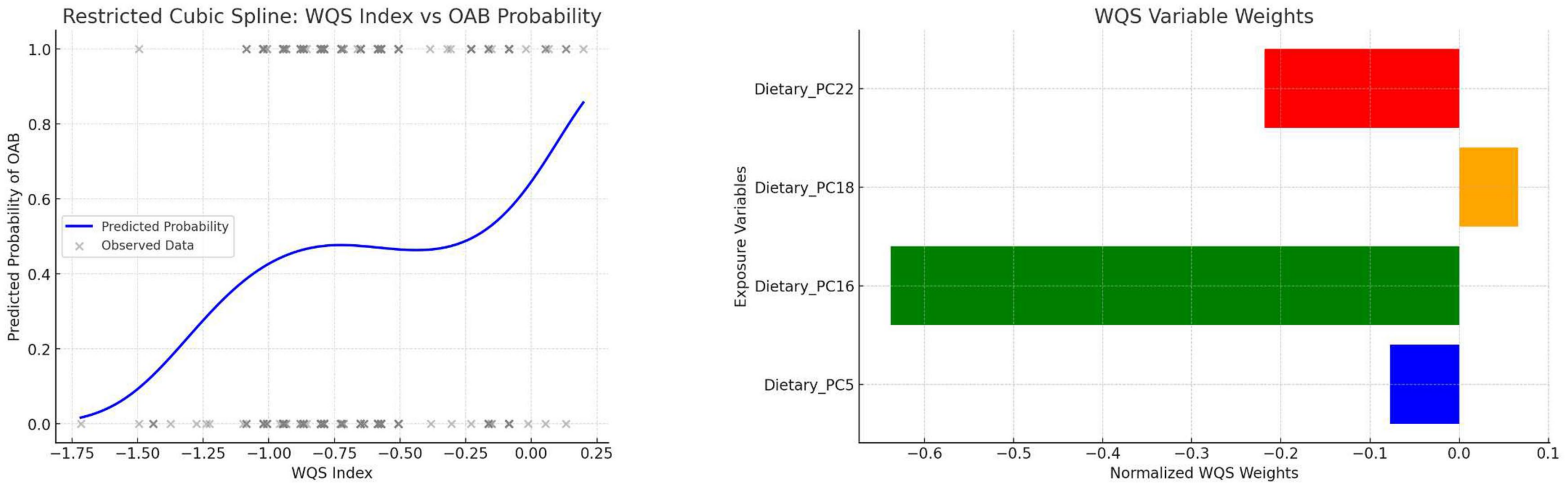
WQS weights and RCS curve of the composite index.

A WQS index was created by combining PC5, PC16, PC18, and PC22 to evaluate the integrated dietary effect on OAB. Key steps included:

Assigning component weights summing to 1.
Estimating contributions via bootstrap resampling.
Assessing nonlinearity between the WQS index and OAB risk using RCS.
Weights and nonlinear trends are visualized in Figure 5.

## 4 Statistical software

Data processing and statistical analyses were conducted using R and Python.

## 5 Ethical statement

This study utilized publicly available, anonymized data from the NHANES database, which requires no additional ethical approval.

## 6 Study results

Four dietary patterns (PC5, PC16, PC18, PC22) were identified as significantly associated with OAB. The nutrient loadings for these patterns are shown in Figure 2, and their characteristics were named based on dominant dietary features:

### 6.1 Antioxidant-rich balanced dietary pattern (PC5)

Logistic Regression: PC5 was negatively associated with OAB (OR < 1, *p* < 0.05).

WQS Model: Minimal contribution to the WQS index, suggesting limited overall impact.

RCS Analysis: OAB risk decreased at low-to-moderate PC5 scores but increased sharply at extreme high scores.

Subgroup Analysis: Stronger protective effects observed in middle-aged participants (≤50–>50 years); no significant associations in obesity or sex subgroups, indicating potential residual confounding.

### 6.2 Diverse low-alcohol dietary pattern (PC16)

Logistic Regression: Significantly negatively associated with OAB (OR < 1, *p* < 0.05).

WQS Model: Largest contributor to the WQS index.

RCS Analysis: Lowest OAB risk at moderate PC16 scores; elevated risk at extreme high/low scores.

Subgroup Analysis: Consistent protective effects across all age groups; nonsignificant trends in obesity and sex subgroups.

### 6.3 Whole grain–high alcohol dietary pattern (PC18)

Logistic Regression: Positively associated with OAB (OR > 1, *p* < 0.05).

WQS Model: Positive contribution to the WQS index.

RCS Analysis: Moderate PC18 scores increased OAB risk, but higher scores reduced risk.

### 6.4 High-fiber low-sugar dietary pattern (PC22)

Logistic Regression: Strong negative association with OAB (OR < 1, p < 0.05).

WQS Model: Negative contribution to the WQS index.

RCS Analysis: Overall trend of reduced OAB risk with higher scores, but extreme low/high scores increased risk.

Subgroup Analysis: Significant protective effects in all subgroups, most pronounced in obesity and female subgroups.

## 7 Discussion

Multiple studies have confirmed a link between dietary health and overactive bladder (OAB). For example, research by Hao et al. (17) suggested that a high dietary health index may have beneficial effects on OAB. This study is not the first to establish an association between dietary patterns and OAB. Previous studies have proposed that certain dietary patterns, such as the Mediterranean diet—rich in whole grains, fruits, vegetables, legumes, nuts, and olive oil—may positively influence lower urinary tract function and reduce OAB symptoms (3). The Mediterranean diet shares some characteristics with the antioxidant-rich balanced dietary pattern (PC5), diverse low-alcohol dietary pattern (PC16), and high-fiber low-sugar dietary pattern (PC22) identified in this study, reflecting an antioxidant-rich and diverse dietary structure. This further supports the notion that healthy dietary patterns may reduce OAB risk.

A study by Barbara Gordon et al. indicated that anti-inflammatory diets (e.g., those rich in dietary fiber, vitamin A, carotenoids, vitamins C, D, E, and certain spices) may improve lower urinary tract dysfunction in women (18). A 2011 study demonstrated that adequate dietary intake of vitamin A, vitamin C, and *β*-carotene was associated with reduced lower urinary tract dysfunction in men (19). These findings further validate the potential benefits of the antioxidant-rich balanced dietary pattern (PC5) and diverse low-alcohol dietary pattern (PC16) for OAB patients.

The whole grain–high alcohol dietary pattern (PC18) includes characteristics of whole grain consumption, which aligns with aspects of the Mediterranean diet and is generally considered beneficial for OAB. However, alcohol intake is a positive loading factor in PC18, and higher PC18 scores correlate with increased alcohol consumption. One study suggested that limiting stimulants like alcohol reduces OAB severity (20), while a 2025 study reported conflicting results but acknowledged that heavy alcohol consumers exhibited higher OAB incidence and severity. This indicates that PC18 is indeed associated with OAB, but its complex dietary composition prevents definitive conclusions about the mechanism.

The protective dietary patterns discussed above are largely linked to mitigating oxidative stress and inflammation, which are recognized mechanisms underlying OAB (21). Regarding specific dietary interventions, one study found that blueberry intake enhanced bladder antioxidant capacity and inhibited bladder remodeling (22), though the exact mechanisms require further investigation. Existing evidence strongly supports that antioxidant-rich and diverse diets alleviate OAB symptoms driven by inflammation and oxidative stress. PC16, characterized by low alcohol and low theobromine intake, suggests that beverage choices may influence OAB development. However, a bibliometric analysis revealed limited and low-quality evidence for associations between OAB and intake of fluids, alcohol, or caffeine (23). No direct studies have explored theobromine’s role in OAB.

Earlier sections discussed protective dietary patterns within normal score ranges. This section examines how extreme scores in these patterns may increase OAB risk. For protective patterns (PC5, PC16, PC22), extreme high scores reflect diets that are antioxidant-rich, diverse, high-fiber, but extremely low-sugar and low-calorie. Current research generally supports that low-sugar, low-calorie diets within moderate ranges protect against OAB. For example, two studies by Maserejian et al. (24) and Maserejian et al. (25) suggested that reducing total calorie intake benefits female OAB patients. However, these studies focused on general populations and did not account for extremes, such as vegan diets. Vegan diets align with antioxidant-rich, diverse, high-fiber, and extreme low-sugar/low-calorie features. One study found that vegans are more prone to muscle loss (26), which correlates with worsened lower urinary tract function (27). This may partially explain the elevated OAB risk at extreme dietary scores, though this observational study cannot establish causality—a key limitation.

Dietary interactions are complex. For example, RCS analysis showed that PC18 acts as a risk factor at low scores but a protective factor at high scores, whereas logistic regression and WQS models identified it as a risk factor overall. This suggests PC18’s relationship with OAB is multifaceted. However, due to sample size limitations and study design, interaction effects were not quantitatively analyzed. Future studies should explore biological mechanisms.

This study has clear limitations. First, data were sourced solely from NHANES, a cross-sectional U.S. population survey without long-term follow-up, precluding causal inferences. Second, dietary data relied on 24-h recall questionnaires (DR1TOT/DR2TOT), which are susceptible to recall bias, social desirability bias (e.g., underreported alcohol intake), and seasonal variations. OAB patients may alter their diets (e.g., reducing fluid intake), introducing reverse causality. Third, PCA with varimax rotation may group conflicting dietary features (e.g., PC18 combines whole grains and alcohol), leading to subjective pattern labeling. Cohort studies are needed to validate individual loadings. Fourth, only three covariates (age, sex, BMI) were included for robustness, potentially limiting subgroup analysis. Additional confounders should be considered. Finally, national, ethnic, and socioeconomic differences—as well as temporal dietary shifts—may influence dietary patterns (28, 29). Generalizing these findings requires validation using databases like the UK National Diet and Nutrition Survey (NDNS) and longitudinal cohort studies to assess long-term dietary impacts on OAB (30).

In this study, we ultimately identified four dietary patterns (PC5, PC16, PC18, and PC22) that exhibited significant associations with OAB. Among these, PC5, PC16, and PC22 demonstrated *p* values well below 0.00074, even after covariate adjustment, meeting the stringent threshold implied by the Bonferroni correction for the 68 principal components. This indicates an extremely low risk of Type I error for these three patterns. Although PC18 also remained statistically significant (*p* < 0.05) following multivariable analysis, its p value exceeded 0.00074, leaving a possibility of false positivity. Nonetheless, because the p value for PC18 remained below 0.05, it warrants further attention. Larger-scale studies replicating this association between PC18 and OAB would help mitigate any residual concerns regarding Type I error. Although the Bonferroni correction effectively controls type I error, the strict threshold may lead to true effects being misjudged as non-significant, thus increasing the risk of type II error (false negatives). Therefore, results with *p*-values between 0.05 and 0.00074 are presented descriptively and are not included in the main conclusions. This limitation should be discussed in detail in the discussion section.

## 8 Conclusion

This study identified three protective dietary patterns (PC5, PC16, PC22) and one dual-effect pattern (PC18). These findings provide critical guidance for dietary interventions in OAB management, emphasizing the importance of balanced and moderate dietary habits for bladder health.

## Data Availability

Publicly available datasets were analyzed in this study. This data can be found here: The datasets analyzed in this study are publicly available from the National Health and Nutrition Examination Survey (NHANES) repository. The data can be accessed directly at the following link: (https://www.cdc.gov/nchs/nhanes/index.htm). NHANES does not use specific accession numbers for datasets, but data from the years 2013–2023 were utilized in this study.
